# Enhancing emotional care: The power of nurse-led interventions in cultivating compassion and family engagement in mental health caregiving in India

**DOI:** 10.6026/973206300212737

**Published:** 2025-08-31

**Authors:** Christina Anthonysamy, Venkatesh Mathan Kumar Vasudevan, Shankar Shanmugam Rajendran, Marudhan Anbalagan, Jayalakshmi Lakshmanan, Maheswari Narayanasamy, Senthilkumar Azhagirisamy

**Affiliations:** 1 Department of Mental Health Nursing, College of Nursing, Madras Medical College, The Tamil Nadu Dr. MGR Medical University, Chennai, Tamil Nadu, India; 2 Department of Mental Health, Institute of Mental Health, Kilpauk, The Tamil Nadu Dr. MGR Medical University, Chennai, Tamil Nadu, India; 3 Department of Child Health Nursing, College of Nursing, Madras Medical College, The Tamil Nadu Dr. MGR Medical University, Chennai, Tamil Nadu, India

**Keywords:** Compassionate care, compassion focused therapy, family engagement

## Abstract

Compassion involves shared suffering and a desire to alleviate it, with family involvement improving patient outcomes, such as reducing relapse and hospital admissions. Therefore, it is of interest to assess the impact of a nurse-led intervention on compassionate care and family engagement among caregivers of mentally ill patients. A quasi-experimental design with 60 samples was used, employing socio-demographic data and structured interviews. Results showed significant improvements in compassionate care and family engagement in the experimental group, while the control group showed minimal changes. Thus, we show the effectiveness of nurse-led interventions in enhancing care and engagement.

## Background:

Mental illnesses are disorders that affect a person's mood, thoughts or behaviours. Serious mental illnesses include a variety of diseases including schizophrenia, bipolar disorder, panic disorder, obsessive-compulsive disorder and major depressive disorder [1]. Family members can be an invaluable resource for individuals dealing with serious mental illnesses. Compassion is defined as a sense of shared suffering, combined with a desire to alleviate or reduce such suffering [2]. Family engagement has been defined as "the process of identifying, enrolling and retaining families in treatment services" [3]. The terms 'carer' and 'caregiver' usually refer to the "substantial, yet 'non-professional' role that individuals in a close relationship have in supporting a person receiving mental health treatment" [4]. Engaging families in patient care supports quality nursing care and patient concordance with treatment [5]. Therefore, it is of interest to determine the effectiveness of nurse-led interventions in enhancing compassionate care and fostering greater family engagement in the caregiving process for mentally ill patients.

## Materials and Methods:

The study was a quasi-experimental design with a non - randomized control group conducted at the acute ward of the Institute of Mental Health in kilpauk, Chennai from March to April 2025.A total of 60 caregivers of mentally ill patient were selected using a non- probability purposive sampling method and divided into an experimental and a control group. Structured interview schedule were used to collect data. Inclusion criteria were caregivers aged 20-60 years and able to communicate in Tamil or English. The permission for conducting the study was obtained from Institutional Ethics Committee (19/11/2024, EC Reg: No:31112024) and Director of Institute of Mental Health. Researcher explained the procedure and written consent was obtained from each participant of the study before starting the data collection.

## Results:

The study predominantly involved most of the samples in the experimental group, most caregivers were aged 41-50 years (46.67%), equally male and female (50%), with primary education (40%) and a family income of ₹5,000-₹10,000 (56.67%). A majority were from nuclear families (80%), urban areas (56.67%), married (73.34%), Hindu (53.33%), and primarily spouses (63.33%) caring for over 5 years (56.67%), with moderate healthcare access (50%). In the control group, caregivers were also mainly aged 41-50 years (56.67%), mostly female (63.33%), graduates (26.67%) with income above ₹10,000 (60%). Most lived in nuclear families (63.33%), urban areas (53.33%), were married (80%), Hindu (40%), and primarily parents (46.67%) caregiving for over 5 years (70%), with moderate to poor healthcare access (36.67%). The participants' level of compassion and family involvement considerably increased. The experimental groups compassionate care improvement score is 14.90%, while the control groups is 1.49%. This also applies to the family engagement score. The experimental group's family engagement gain score is 53.06%, while the control groups are 1.93%. For family Engagement score Homemakers showed 100% high engagement, while unskilled workers had predominantly moderate scores. Caregivers with easy access to healthcare and 3-5 years of caregiving experience reported higher engagement. For compassionate care, significant factors included gender, monthly income, and type of family. Females, caregivers earning > Rs. 10,000 and those from joint families had higher compassionate care scores.

Table 1 (see PDF) presents a comparison of family engagement scores between the experimental and control groups. At the pre-test, the mean scores for both groups were very similar, with the experimental group having a mean of 53.06 and the control group a mean of 53.44. The difference was not statistically significant, as indicated by a t-value of 0.25 and a p-value of 0.81, which is not significant (NS). However, at the post-test, the experimental group showed a significant improvement, with a mean score of 73.33, compared to the control group, which had a mean of 55.56. This difference was statistically significant, with a t-value of 12.28 and a p-value of 0.001, indicating a strong result (S). Table 2 (see PDF) compares compassionate care scores between the experimental and control groups. Similar to Table 1 (see PDF), at the pre-test, both groups had nearly identical mean scores (experimental group: 50.27, control group: 49.77), with no significant difference (t = 0.30, p = 0.77). However, at the post-test, the scores diverged significantly, with the experimental group showing a mean score of 30.9 and the control group having a mean of 47.83. This difference was statistically significant, as evidenced by a t-value of 17.65 and a p-value of 0.001, indicating a significant change (S).

## Discussion:

The outcomes of this study confirm that nurse-led interventions significantly enhance compassionate care and family engagement among caregivers. This is supported by similar randomized trials, which demonstrated improved psychological outcomes and caregiver well-being following compassion-based interventions, affirming the present study's effectiveness. These findings emphasize the positive impact of nurse-led interventions in fostering compassionate care and family engagement. Enhanced self-compassion significantly contributes to better coping, recovery, and overall well-being, as supported by related studies highlighting its mediating and transformative role in caregiving. The findings of the present study are supported by a quasi-experimental investigation conducted by Zaki *et al.* (2025) [6], which evaluated the impact of a Compassion-Based Intervention Program on coping strategies among 60 family caregivers of individuals with bipolar disorder in Egypt. Before the intervention, 62% of participants showed low levels of self-compassion. However, post-intervention assessments revealed a significant improvement, with 54% reporting high self-compassion (p < .01), thus affirming the outcomes observed in this study. These results echo those of Reinhard *et al.* (2014) [7], who identified the importance of structured family involvement in the treatment of mental illness, reinforcing the need for caregiver support in enhancing patient outcomes. Ong *et al.* (2021) [4] also highlighted the role of family engagement in managing mental illness, with your findings of improved family involvement further supporting this notion. Additionally, Sengupta and Saxena (2024) [2] emphasized the centrality of compassion in mental healthcare, which is reflected in your study's outcomes where compassionate care significantly improved among caregivers. Overall, the results of our research substantiate and extend the findings of these prior studies, demonstrating the efficacy of nurse-led interventions in fostering compassionate care and active family involvement in mental health caregiving.

## Conclusion:

Nurse-led interventions can significantly improve compassionate care and family engagement among caregivers of mentally ill patients. By providing knowledge and emotional support, nurses empower caregivers, fostering a collaborative environment essential for recovery. Thus, we show the need to integrate structured caregiver support into mental health services to enhance both caregiver well-being and patient outcomes.
